# Characterization and Phylogenetic Analysis of the Chloroplast Genome of *Elaeagnus oxycarpa* Schltdl

**DOI:** 10.3390/biology15070590

**Published:** 2026-04-07

**Authors:** Kaidiriye Yusupu, Qiyu Gu, Boqiang Wei, Hui Geng, Li Xiong

**Affiliations:** 1 Key Laboratory of Biological Resources and Ecology of Pamirs Plateau in Xinjiang Uygur Autonomous Region, College of Life and Geographic Sciences, Kashi University, Kashi 844000, China; 2 Key Laboratory of Pesticide and Chemical Biology of Ministry of Education, Hubei Key Laboratory of Genetic Regulation and Integrative Biology, School of Life Sciences, Central China Normal University, Wuhan 430079, China

**Keywords:** *Elaeagnus oxycarpa*, chloroplast genome, genomic structure, codon usage bias, simple sequence repeats (SSRs), comparative genomics, phylogenetic analysis

## Abstract

Background: *Elaeagnus oxycarpa* Schltdl. (Elaeagnaceae) is a vital sand-fixing tree species in the arid, semi-arid, and desert regions of China, holding significant ecological and economic value. However, its chloroplast (cp) genome has not been previously characterized. Results: In this study, we sequenced the complete cp genome of *Elaeagnus oxycarpa* using Illumina high-throughput sequencing technology. We performed de novo assembly, annotation, and comparative genomic and phylogenetic analyses with six other Elaeagnaceae species. The results revealed a typical quadripartite structure for the *E. oxycarpa* cp genome, with a total length of 150,567 bp and a GC content of 36.90%. Annotation identified 132 genes, comprising 86 protein-coding genes (PCGs), 38 tRNA genes, and 8 rRNA genes. Codon usage bias analysis indicated a preference for A/U endings, with leucine codons being the most frequent (9.5%). Additionally, 77 simple sequence repeat (SSR) loci were detected, predominantly mononucleotide repeats (71.4%). Comparative genomic analysis demonstrated high sequence conservation among the seven *Elaeagnus* species, with nucleotide variations primarily concentrated in non-coding regions and intergenic spacers of genes such as *rps*16, *ycf*1, and *trn*C-GCA. These variable regions and SSR loci represent valuable molecular markers for future population genetics and species identification studies on *Elaeagnus*. Phylogenetic analysis strongly supported the notion that *E. oxycarpa* and *Elaeagnus angustifolia* form a sister clade, indicating their close genetic relationship. Conclusions: Our findings provide crucial genomic resources and a theoretical foundation for the species identification and elucidation of the evolutionary history of Elaeagnaceae.

## Simple Summary

*Elaeagnus oxycarpa* is a key tree species for combating desertification in the arid regions of China, holding great ecological and economic value. However, as its complete chloroplast (cp) genome has not been investigated, in this study, we decoded its full cp genome. The genome is circular, 150,567 base pairs in length, and contains 132 functional genes. We analyzed its genetic code usage, which shows a preference for A/U nucleotides, and identified 77 simple sequence repeats. The genome structure is highly conserved compared to other related species. A family tree based on genome data strongly supports the notion that *E. oxycarpa* is most closely related to *Elaeagnus angustifolia*. This research provides the first complete chloroplast genome resource for *E. oxycarpa*, which will prove valuable in future studies investigating its conservation, species identification, and adaptation to harsh environments.

## 1. Introduction

The genus *Elaeagnus* (Elaeagnaceae) comprises plants renowned for their dual ecological and economic importance. With over 90 species distributed across temperate and subtropical regions of Asia, Europe, and North America, approximately 50 species are native to China, predominantly found in the Southwest, Northwest, and North of the country [1,2]. Species within this genus are primarily deciduous or evergreen trees and shrubs, characterized by exceptional adaptability to environmental stresses such as drought, salinity, alkalinity, and wind erosion [3]. These traits make them keystone species in fragile ecosystems, where they play a critical role in combating desertification, stabilizing sand dunes, and maintaining regional ecological balance. Economically, their fruits are rich in vitamins, fructose, and amino acids, whether consumed fresh or processed into jams and dried foods. Additionally, leaves and bark of some species contain bioactive compounds with medicinal properties, traditionally used to alleviate cough and diarrhea, indicating considerable pharmaceutical potential [4,5,6,7,8]. To date, several chloroplast genomes within the genus *Elaeagnus* have been sequenced and analyzed, including *E. angustifolia* [1], *E. mollis* [2], *E. oldhamii* [3], and others [4,5], providing insights into the genomic structure and phylogenetic relationships of the genus. *Elaeagnus oxycarpa* Schltdl., a deciduous tree within the genus, is a cornerstone species for afforestation and sand fixation in the arid, semi-arid, and desert regions of northwestern China [9]. Its exceptional ability to thrive in nutrient-poor sandy soils and withstand severe wind erosion makes it an invaluable natural barrier against desert expansion. Beyond its paramount ecological role in soil conservation, *E. oxycarpa* also offers significant economic benefits through its nutritious and medicinally promising fruits [10,11,12]. To date, most research on *E. oxycarpa* has focused on its biological characteristics, ecological functions, and preliminary phytochemical analyses [10,11,12]. However, in-depth genomic studies, particularly on its chloroplast genome, are lacking for this specific species, with no cp genome data available in the NCBI database prior to this study.

Chloroplasts—essential organelles for photosynthesis—possess semi-autonomous genomes characterized by a stable structure, high copy number, and moderate evolutionary rate. These features make them ideal molecular markers for phylogenetic studies across various taxonomic levels [13,14,15,16]. The cp genomes of higher plants are typically double-stranded, circular DNA molecules that range from 120 to 160 kb, exhibiting a characteristic quadripartite structure comprising a large single-copy (LSC) region, a small single-copy (SSC) region, and a pair of inverted repeat (IRa/IRb) regions [15]. Understanding the cp genome is therefore crucial not only for elucidating evolutionary relationships but also for uncovering potential genetic adaptations to environmental stresses. The absence of cp genome data for *E. oxycarpa* has hindered a precise determination of its evolutionary position within the genus, thereby limiting a comprehensive understanding of *Elaeagnus* classification and evolutionary mechanisms.

This study aimed to sequence, assemble, and annotate the complete cp genome of *E. oxycarpa* to characterize its structural features, gene composition, codon usage bias, SSR loci, and sequence divergence. Through comparative genomic and phylogenetic analyses with other Elaeagnaceae species, we sought to clarify the phylogenetic relationship of *E. oxycarpa*. Our findings fill a critical knowledge gap in the chloroplast genomics of this ecologically vital species and provide fundamental genetic resources for future studies on its genetic diversity, population evolution, and molecular breeding, and, importantly, these resources help understand the genomic basis of this species’ remarkable ecological adaptations.

## 2. Materials and Methods

### 2.1. Plant Material and Data Acquisition

Fresh leaves of *E. oxycarpa* were collected from Altay County, Xinjiang Uygur Autonomous Region by researchers from the Molecular Biology Experiment Center, Germplasm Bank of Wild Species in Southwest China, and the National Wild Plant Germplasm Resource Center, Kunming Institute of Botany, Chinese Academy of Sciences (cstr.cn/31121.02.GBOWS). The plant material of *Elaeagnus oxycarpa* used in this study was formally identified by Prof. Cheng Liu, Kunming Institute of Botany, Chinese Academy of Sciences. Voucher specimens (No. 16CS12328) were deposited at the same institute.

### 2.2. DNA Extraction, Genome Sequencing, Assembly, and Annotation

Total genomic DNA was extracted from flash-frozen leaf samples using a Plant Genomic DNA Kit (Tiangen Biotech, Beijing, China) according to the manufacturer’s instructions. DNA quality was assessed via 1% agarose gel electrophoresis and spectrophotometry. Additionally, complete cp genome sequences of 12 related species were downloaded from the NCBI database for comparative analysis (see Table S1 for accession numbers).

Paired-end sequencing libraries were constructed and sequenced on an Illumina HiSeq 2500 platform at Guangzhou Genedenovo Biotechnology Co., Ltd., Guangzhou, China, generating approximately 5.2 Gb of raw data. Raw reads were quality-trimmed using Trimmomatic v.0.39 [17], resulting in 5 Gb of clean data. The cp genome was assembled de novo using GetOrganelle v.1.7.5 [18], with parameters -R 30 -k 21,45,65,85,105,127 -F embplant_pt. The assembly’s structural integrity was verified using Bandage v.0.8.1 [19]. Genome annotation was performed using the online tool GeSeq (https://chlorobox.mpimp-golm.mpg.de/geseq.html, accessed on 31 March 2026) [20], followed by manual correction of gene boundaries, introns/exons, and stop codons in Geneious Prime v.2022.1.1 [21]. The annotated genome map was visualized and optimized using OGDRAW v.1.3 [20].

### 2.3. Codon Usage Bias and SSR Analysis

Codon usage bias was analyzed using CodonW v.1.4.2 [18] which calculated the Relative Synonymous Codon Usage (RSCU) values. RSCU >1 indicates positive codon usage bias, RSCU <1 indicates negative bias, and RSCU = 1 indicates no bias. Simple sequence repeats (SSRs) were identified using MISA v.2.1 [22], with minimum repeat thresholds set to 10 (mono-), 5 (di-), 4 (tri-), and 3 (tetra-, penta-, and hexa-nucleotide).

### 2.4. IR Boundary Shifts and Comparative Genomic Analysis

The IRscope online tool [23] was used to analyze the contraction/expansion of IR boundaries and the distribution of adjacent genes, comparing *E. oxycarpa* with six congeneric species. Whole-genome alignment and sequence divergence analysis were performed using Shuffle-LAGAN mode in mVISTA [24], with the *E. oxycarpa* cp genome serving as the reference.

### 2.5. Phylogenetic Analysis

Thirteen complete cp genome sequences, including *E. oxycarpa* and twelve related species (with *Rosa cymosa* as the outgroup), were aligned using MAFFT v.7 [25]. Poorly aligned regions were removed using Gblocks v.0.91b [26]. The optimal nucleotide substitution model (GTR+F+R4) was selected by ModelFinder [27] based on the Akaike Information Criterion (AIC). A maximum likelihood (ML) phylogenetic tree was constructed using IQ-TREE v.2.1.4 [28], with branch support assessed by 1000 ultrafast bootstrap replicates [29]. The tree was visualized using FigTree v.1.4.3.

## 3. Results

### 3.1. General Features of the Chloroplast Genome

**Figure 1:**
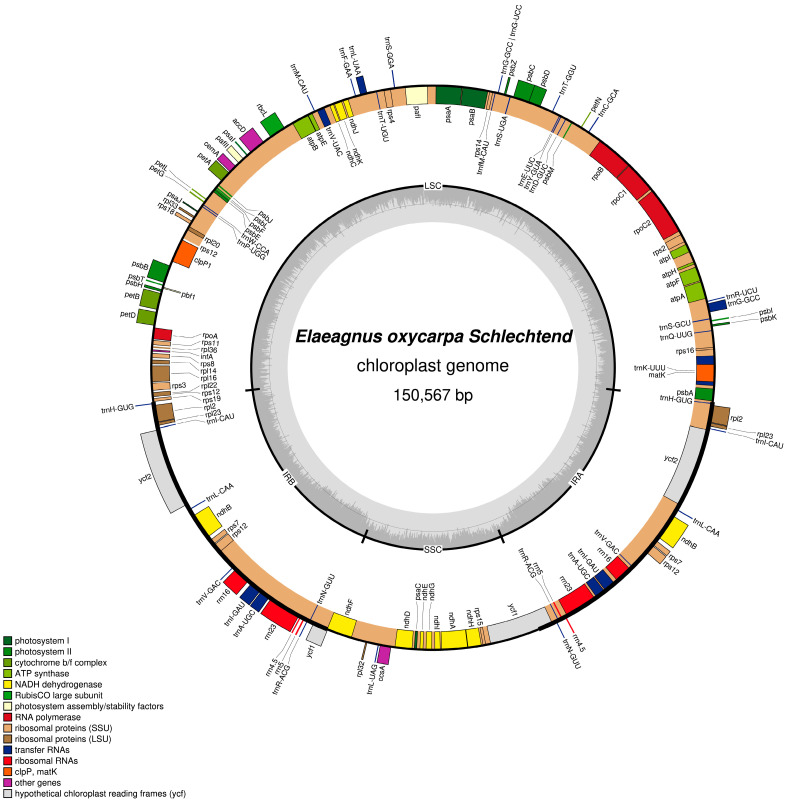
Chloroplast genome ring map of *Elaeagnus oxycarpa*.

The complete cp genome of *E. oxycarpa* is a circular DNA molecule of 150,567 bp, exhibiting the typical quadripartite structure: a large single-copy region (LSC: 81,133 bp), a small single-copy region (SSC: 18,446 bp), and two inverted repeat regions (IR: 25,494 bp each) (Figure 1, Table 1). The overall GC content was 36.90%, with the IR regions exhibiting the highest GC content (42.70%), followed by the LSC (34.90%) and SSC (30.20%) regions (Table 1). Annotation identified 132 unique genes, including 86 PCGs, 38 tRNAs, and 8 rRNAs (Table 2).

### 3.2. Codon Usage Bias

**Figure 2:**
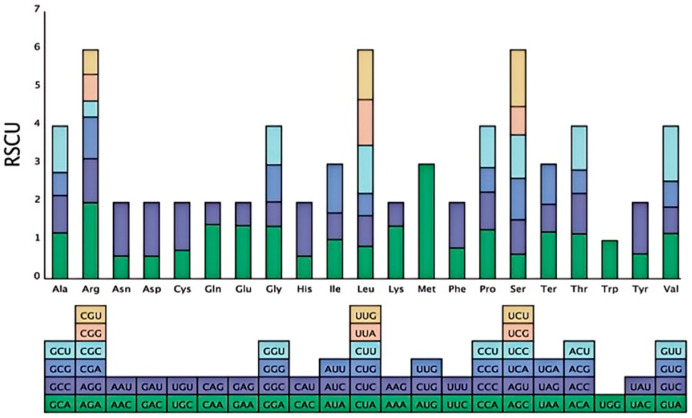
RSCU (Relative Synonymous Codon Usage) analysis of amino acids of *Elaeagnus oxycarpa*.

Analysis of 50,189 codons revealed that leucine (Leu; 9.5%) was the most abundant amino acid. RSCU analysis identified 35 codons with RSCU > 1, indicating a strong preference for A/U endings at the third codon position (Figure 2). For instance, the alanine codons GCA (RSCU = 1.20) and GCU (RSCU = 1.21) and the asparagine codon AAU (RSCU = 1.40) were preferentially used.

### 3.3. SSR Loci Analysis

Seventy-seven SSR loci were identified (Table 3), predominantly mononucleotide repeats (55, 71.43%), consisting mainly of A/T repeats. Dinucleotide (11), trinucleotide (6), tetranucleotide (2), pentanucleotide (2), and hexanucleotide (1) repeats were also detected. Most SSRs were located in intergenic spacer (IGS) regions, and a few were within genes like *trn*K-UUU, *clp*P, and *ycf*1 (Table 4).

### 3.4. IR Boundary Analysis

**Figure 3:**
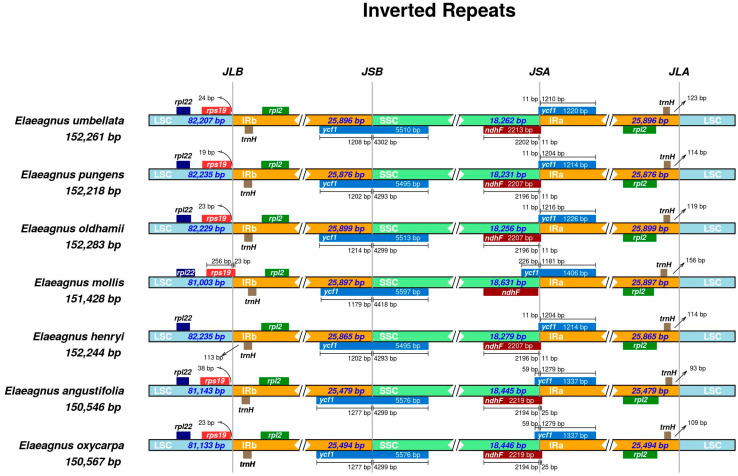
IR boundary diagram of chloroplast genome of seven species of *Elaeagnus*.

IR boundary analysis among seven *Elaeagnu*s species revealed highly conserved structures and gene compositions (Figure 3). Genome lengths varied from 150,546 bp (*E. angustifolia*) to 152,283 bp (*E. oldhamii*), with corresponding IR lengths ranging from 25,479 bp to 25,899 bp. The *rps*19 gene was absent at the LSC/IRb junction only in *E. henryi*. The *ycf*1 gene spanned the IRb/SSC and IRa/SSC junctions, with fragment lengths varying due to IR expansion/contraction.

### 3.5. Comparative Genomics

**Figure 4:**
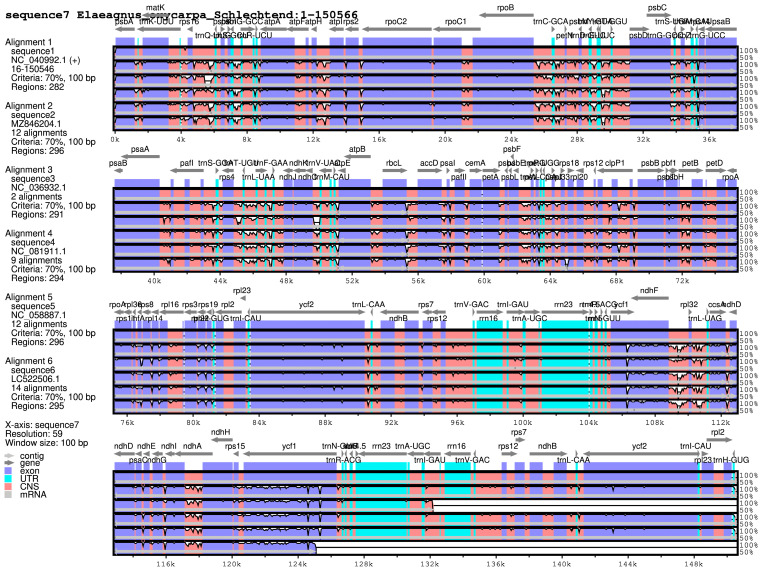
Chloroplast genome comparison of *Elaeagnus oxycarpa* and six other species of the *Elaeagnus* genus. Note: UTRs represent untranslated regions; CNS stands for non-coding sequence.

Whole-genome comparison revealed high sequence identity among the seven *Elaeagnus* species (Figure 4). Highly divergent regions were primarily located in non-coding sequences and within specific genes like *rps*16, *trn*C-GCA, and *ycf*1.

### 3.6. Phylogenetic Relationships

**Figure 5:**
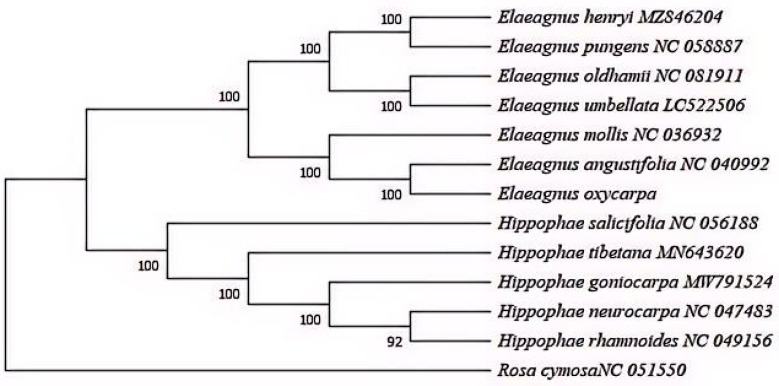
Phylogenetic tree constructed via the ML method based on the whole chloroplast genome of 13 species.

Phylogenetic analysis yielded a well-supported phylogenetic tree (Figure 5) that clearly separates *Elaeagnus* and *Hippophae* species, with *Rosa cymosa* as the outgroup. Within *Elaeagnus*, *E. oxycarpa* and *E. angustifolia* formed a strongly supported sister clade, with *E. mollis* as their closest relative.

## 4. Discussion

This study presents the first complete cp genome sequence of *Elaeagnus oxycarpa*. Its quadripartite structure is consistent with typical angiosperm cp genomes and previously reported Elaeagnaceae species [28,29,30,31], which underscores the structural conservation of cp genomes driven by functional constraints on essential genes involved in photosynthesis and energy metabolism [15]. However, interspecific divergence was evident in our analysis. The GC content (36.90%) was lower than that of *Hippophae* species (37.8–38.2%) and slightly lower than *E. angustifolia* [30]. This lower GC content might result from synergistic effects of evolutionary forces. Genetic drift in small, fragmented wild populations of *E. oxycarpa* could favor the fixation of GC→AT mutations. Additionally, natural selection might favor lower GC content in arid high-temperature habitats, as it reduces DNA melting temperature, which potentially facilitates rapid gene transcription under stress [31], aligning with *E. oxycarpa*’s adaptation to Northwestern China’s arid regions. However, we note that this correlation, while intriguing, requires further population-level and functional studies to establish a causal link. Comparative analysis with other arid-adapted *Elaeagnus* species (e.g., *E. angustifolia* from similar habitats) would help clarify if this genomic feature is a common adaptive trait or species-specific.

The gene repertoire (132 genes) is typical of higher plant cp genomes [5,15]. The pronounced A/U bias in codon usage (68.3% at the third position), which is also observed in other *Elaeagnus* species such as *E. angustifolia* [30] and *E. oldhamii* [3], though slightly more pronounced in *E. oxycarpa*, may enhance translational efficiency by matching abundant tRNAs, which is crucial for the rapid synthesis of photosynthetic proteins (e.g., Rubisco) in variable environments [13,32]. Mutational pressure and high expression levels of photosynthetic genes likely contribute to this bias [33].

The 77 identified SSR loci, mostly mononucleotide repeats, represent valuable molecular markers for assessing genetic diversity, population structure, gene flow, cultivar identification, and molecular breeding in *E. oxycarpa* [34,35]. Comparative analysis of these loci across Elaeagnaceae can further clarify phylogenetic relationships [36].

The robust phylogenetic tree confirms the close relationship between *E. oxycarpa* and *E. angustifolia*, consistent with morphological and ecological similarities, thereby supporting the current taxonomic framework [37]. This provides a solid basis for future studies on Elaeagnaceae evolution, including divergence time estimation using molecular clocks.

It is noteworthy that *E. oxycarpa* Schltdl. has sometimes been treated as a synonym or a variant of *E. angustifolia* L. in some taxonomic treatments (e.g., Plants of the World Online). Our phylogenetic results, showing them as sister species with strong support, are consistent with a very close relationship, supporting either a distinct species status or a very recent divergence within the *E. angustifolia* complex. The genomic resources provided here will aid in resolving this taxonomic nuance through more extensive population-level studies.

## 5. Conclusions

We successfully sequenced, assembled, and annotated the complete chloroplast genome of *Elaeagnus oxycarpa*. The genome exhibits a typical structure but features a lower GC content and a strong A/U codon usage bias, potentially linked to its adaptation to arid environments. The identified SSRs and highly variable regions (e.g., *rps*16, *trn*C-GCA, and *ycf*1) provide powerful tools for species identification and population genetics. Phylogenetic analysis firmly establishes *E. oxycarpa* and *E. angustifolia* as sister species. This study provides essential genomic resources for future research on the conservation, molecular breeding, and adaptive evolution of this ecologically crucial species.

## Figures and Tables

**Table 1:** Base composition of chloroplast genome of *Elaeagnus oxycarpa*.

Area	A /%	C /%	G /%	T/U /%	Length/bp	GC /%
LSC	31.70	17.90	17.00	33.40	81,133	34.90
SSC	34.90	15.60	14.60	34.90	18,446	30.20
IRa	28.46	22.22	20.46	28.86	25,494	42.70
IRb	28.86	20.46	22.22	28.46	25,494	42.70
Total	31.10	18.80	18.20	32.00	150,567	36.90

Note: 1. SSC, small single copy; 2. LSC, large single copy; 3. IRa/IRb, inverted repeat.

**Table 2:** Chloroplast genome annotation information of *Elaeagnus oxycarpa*.

Category of Genes	Grouping of Genes	Name of Gene
Genes for Photosynthesis	Subunits of photosystem I	*psaB*, *psaA*, *psaI*, *psaC*, *psaJ*
	Subunits of photosystem II	*psbA*, *psbD*, *psbN*, *psbE*, *psbH*, *psbI*, *psbC*, *psbF*, *psbJ*, *psbK*, *psbL*, *psbB*, *psbM*, *psbT*, *psbZ*
	Subunit of cytochrome b/f complex	*petL*, *petB* *, *petD* *, *PetA*, *petG*, *petN*
	Subunits of ATP synthase	*atpA*, *atpF* *, *atpB*, *atpH*, *atpI*, *atpE*
	Subunits of NADH dehydrogenase	*ndhA* *, *ndhC*, *ndhI*, *ndhB* *^#^, *ndhE*, *ndhF*, *ndhH*, *ndhD*, *ndhJ*, *ndhG*, *ndhK*
	Large subunit of Rubisco	*rbcL*
	ATP-dependent protease subunit P	*clpP* **
Self-replicating genes	DNA dependent RNA polymerase	*rpoB*, *rpoA*, *rpoC1* *, *rpoC2*
	Small subunit of ribosome	*rps2*, *rps3*, *rps18*, *rps7* *, *rps11*, *rps12* *^#^, *rps14*, *rps4*, *rps15*, *rps16*, *rps8*, *rps19*
	Large subunit of ribosome	*rpl2* *^#^, *rpl33*, *rpl16* *, *rpl20*, *rpl23* ^#^, *rpl32*, *rpl14*, *rpl22*, *rpl36*
	Ribosomal RNAs	*rrn4.5(x2)*, *rrn5(x2)*, *rrn16(x2)*, *rrn23(x2)*
	Transfer RNAs	*trnA-UGC(x2)*, *trnC-GCA*, *trnD-GUC*, *trnE-UUC*, *trnF-GAA*, *trnfM-CAU*, *trnG-GCC*, *trnG-UCC*, *trnH-GUG*, *trnI-CAU(x2)*, *trnI-GAU(x2)*, *trnK-UUU*, *trnL-CAA(x2)*, *trnL-UAA*, *trnL-UAG*, *trnM-CAU*, *trnN-GUU(x2)*, *trnP-UGG*, *trnQ-UUG*, *trnR-ACG(x2)*, *trnR-UCU*, *trnS-GCU*, *trnS-GGA*, *trnS-UGA*, *trnT-GGU*, *trnT-UGU*, *trnV-GAC(x2)*, *trnV-UAC*, *trnW-CCA*, *trnY-GUA*
Other Genes	Maturase K	*matK*
	Envelope membrane protein	*cemA*
	Acetyl-CoA carboxylase subunit	*accD*
	C-type cytochrome synthesis gene	*ccsA*
Genes of Unknown Function	Conserved open reading frames	*ycf1(x2)*, *ycf2(x2)*, *ycf3* *, *ycf4*

Note: Genes containing introns are marked with one (*) for one intron or two (**) for two introns, (^#^) indicates a trans-spliced gene and (*x2*) indicates genes duplicated in the IR regions.

**Table 3:** Types of simple sequence repeats (SSRs) in the chloroplast genome of *Elaeagnus oxycarpa*.

Repeat Unit	Type	Number	Largest Repeat
1	A	19	16
	T	36	18
2	AT	5	5
	TA	6	5
3	TCT	1	4
	TAT	1	4
	AAT	2	4
	CTT	1	4
	AAG	1	4
4	TTTA	1	3
	TCTT	1	3
5	TATTA	1	3
	TAATA	1	3
6	ATCTAT	1	3
Total	14	77	-

**Table 4:** Simple repeat sequence (SSR) information of the chloroplast genome of *Elaeagnus oxycarpa*.

NO.	SSR Type	SSR	Size	Start	End	Location
1	p1	(T)13	13	1307	1319	IGS
2	p1	(A)14	14	1427	1440	trnK-UUU *
3	p3	(TCT)4	12	1813	1824	trnK-UUU *-matK
4	p1	(T)11	11	1949	1959	trnK-UUU *-matK
5	p6	(ATCTAT)3	18	5730	5747	IGS
6	c	(AT)5agagagagaataaatctatatctatctaacagactatggattctattatttcatagaatatctaatagaatctaaatag(AT)5	99	5864	5962	IGS
7	p1	(A)16	16	6965	6980	IGS
8	c	(TA)5aaaaaagaaaaaacc(T)13atttgtccccagggactctttcatttccacggcttggcctgggcaggcccagccgggcttcttttgttctaacaaatcgtaataa(T)11catattttttttattattttattgctattctatttaccgatattttactgaaaaagataaagaaagatagagttcattttggatttttggacaatg(T)10	240	7193	7432	IGS
9	p1	(T)10	10	8234	8243	trnG-UCC *
10	p1	(T)10	10	12,894	12,903	IGS
11	p1	(A)11	11	13,828	13,838	IGS
12	p1	(T)10	10	14,761	14,770	IGS
13	p1	(A)11	11	14,982	14,992	IGS
14	p2	(TA)5	10	18,406	18,415	rpoC2
15	p1	(T)11	11	19,457	19,467	rpoC1 *
16	p1	(T)10	10	24,703	24,712	rpoB
17	p1	(A)11	11	26,199	26,209	IGS
18	p2	(TA)5	10	29,988	29,997	IGS
19	p1	(T)11	11	30,477	30,487	IGS
20	c	(A)10tagggatcacttgtttcttgaacagttctt(A)12	52	30,914	30,965	IGS
21	p1	(T)11	11	33,774	33,784	IGS
22	p1	(A)10	10	34,121	34,130	IGS
23	p1	(T)10	10	34,624	34,633	IGS
24	p1	(A)12	12	41,002	41,013	IGS
25	p4	(TTTA)3	12	41,732	41,743	ycf3 *
26	p1	(T)12	12	43,703	43,714	IGS
27	p2	(TA)5	10	45,009	45,018	IGS
28	p2	(AT)5	10	45,546	45,555	IGS
29	p1	(T)10	10	45,681	45,690	IGS
30	c	(AT)5taatatgtatatctatacatattgaatttcggatacagaaatgataaaatcttttatgattgggcaaaatatgaatttccgatag(A)10	105	45,931	46,035	IGS
31	c	(T)10attgatatgaaaaatgaaaaaattgttgtgaatcgattcacatctg(A)11	67	46,556	46,622	trnL-UAA *
32	p2	(AT)5	10	50,054	50,063	IGS
33	p1	(T)10	10	51,090	51,099	IGS
34	p1	(T)11	11	53,033	53,043	atpB
35	p1	(A)13	13	53,622	53,634	IGS
36	p3	(TAT)4	12	57,648	57,659	IGS
37	p1	(A)11	11	58,148	58,158	IGS
38	p1	(T)11	11	63,899	63,909	IGS
39	p2	(TA)5	10	64,027	64,036	IGS
40	p1	(T)11	11	64,325	64,335	IGS
41	P3	(AAT)4	12	64,449	64,460	IGS
42	p1	(T)13	13	66,140	66,152	IGS
43	c	(T)18gaaaaat(A)11ttctcatatcgaattcgaagtgccatgctattattactcaatattcatatagcgcgaaggcatagtcctctttttgtctctcaaat(A)13	135	67,610	67,744	clpP *
44	p1	(A)11	11	68,122	68,132	clpP *
45	c	(T)10aggtttatgctctactccgagtaaagatccgcccgatttggatttgcacatatagaacaaatgccccaataccactttcatacgactttcccc(T)10	113	68,381	68,493	clpP *
46	p1	(A)13	13	76,591	76,603	IGS
47	c	(TCTT)3tta(T)11	26	76,904	76,929	IGS
48	p1	(T)10	10	77,320	77,329	rps8
49	p1	(T)17	17	77,439	77,455	IGS
50	c	(T)10atctg(T)14	29	77,907	77,935	IGS
51	p1	(A)11	11	78,537	78,547	rpl16 *
52	p1	(T)11	11	80,690	80,700	rpl22
53	p1	(T)10	10	81,127	81,136	IGS
54	p3	(CTT)4	12	87,545	87,556	ycf2
55	p5	(TATTA)3	15	96,041	96,055	IGS
56	p1	(T)10	10	109,257	109,266	IGS
57	c	(T)10atttgataaaaaaaagtttttttgaagtgtggcaatgttacaattatgataatgctacaaaaatgtttac(TA)5	90	110,536	110,625	IGS
58	p1	(A)12	12	120,095	120,106	IGS
59	p3	(AAT)4	12	120,448	120,459	IGS
60	p1	(T)12	12	121,888	121,899	ycf1
61	p1	(T)12	12	123,050	123,061	ycf1
62	p1	(T)14	14	123,219	123,232	ycf1
63	p5	(TAATA)3	15	135,646	135,660	IGS
64	p3	(AAG)4	12	144,145	144,156	ycf2

Note: The number in p1/p2 indicates the number of bases constituting the motif, and p represents a single SSR type; IGS represents the intergenic region; c represents the compound SSR type; * represents an intron.

## Data Availability

All data generated or analyzed during this study are included in this published article and its Supplementary Information Files.

## Supplementary Materials

The following supporting information can be downloaded at https://www.mdpi.com/article/10.3390/biology15070590/s1. Table S1: NCBI accession numbers for 12 species.

## Abbreviations

The following abbreviations are used in this manuscript:

AIC	Akaike Information Criterion
ATP	Adenosine Triphosphate
CNS	Conserved Non-coding Sequence
cp	Chloroplast
IGS	Intergenic Spacer
IR	Inverted Repeat
Leu	Leucine
LSC	Large Single-Copy
ML	Maximum Likelihood
NADH	Nicotinamide Adenine Dinucleotide Hydrogen
PCGs	Protein-Coding Genes
RSCU	Relative Synonymous Codon Usage
SSC	Small Single-Copy
SSR	Simple Sequence Repeat
UTRs	Untranslated Regions
