# Supplementary material for: Characterization and Phylogenetic Analysis of the Chloroplast Genome of Elaeagnus oxycarpa Schltdl
Source: Biology (Basel). 2026 Apr 7;15(7):590. doi: 10.3390/biology15070590 (PMC13072164; doi:10.3390/biology15070590)
Supplement: Supplementary file 1 [file biology-15-00590-s001.zip › biology-4233507-supplementary.pdf]

Table S1 NCBI accession numbers for 12 species

Species name	NCBI accession number for the chloroplast genome
Elaeagnus henryi	MZ846204.1
Elaeagnus pungens	NC_058887.1
Elaeagnus oldhamii	NC_081911.1
Elaeagnus umbellata	LC522506.1
Elaeagnus mollis	NC_036932.1
Elaeagnus angustifolia	NC_040992.1
Hippophae salicifolia	NC_047483.1
Hippophae tibetana	NC_056188.1
Hippophae goniocarpa	MW791524.1
Hippophae neurocarpa	NC_047483.1
Hippophae rhamnoides	NC_047483.1
Rosa cymosa	NC_056188.1
